# Role of Ultrasound and Photoacoustic Imaging in Photodynamic Therapy for Cancer

**DOI:** 10.1111/php.13217

**Published:** 2020-03-05

**Authors:** Scott C. Hester, Maju Kuriakose, Christopher D. Nguyen, Srivalleesha Mallidi

**Affiliations:** ^1^ Department of Biomedical Engineering Tufts University Medford MA

## Abstract

Photodynamic therapy (PDT) is a phototoxic treatment with high spatial and temporal control and has shown tremendous promise in the management of cancer due to its high efficacy and minimal side effects. PDT efficacy is dictated by a complex relationship between dosimetry parameters such as the concentration of the photosensitizer at the tumor site, its spatial localization (intracellular or extracellular), light dose and distribution, oxygen distribution and concentration, and the heterogeneity of the inter‐ and intratumoral microenvironment. Studying and characterizing these parameters, along with monitoring tumor heterogeneity pre‐ and post‐PDT, provides essential data for predicting therapeutic response and the design of subsequent therapies. In this review, we elucidate the role of ultrasound (US) and photoacoustic imaging in improving PDT‐mediated outcomes in cancer—from tracking photosensitizer uptake and vascular destruction, to measuring oxygenation dynamics and the overall evaluation of tumor responses. We also present recent advances in multifunctional theranostic nanomaterials that can improve either US or photoacoustic imaging contrast, as well as deliver photosensitizers specifically to tumors. Given the wide availability, low‐cost, portability and nonionizing nature of US and photoacoustic imaging, together with their capabilities of providing multiparametric morphological and functional information, these technologies are thusly inimitable when deployed in conjunction with PDT.

## Graphical abstract

Here we review the role of ultrasound (US) and photoacoustic imaging in improving photodynamic therapy (PDT)‐mediated outcomes in cancer. The utility of these techniques in providing multiparametric morphological and functional information, particularly on tracking photosensitizer uptake and vascular destruction, and measuring oxygenation dynamics and the overall evaluation of tumor response, is demonstrated. We also present recent advances in multifunctional theranostic nanomaterials that improve either US or photoacoustic imaging contrast, as well as specifically deliver photosensitizers to tumors. Given the wide availability, low‐cost, portability and nonionizing nature of US and photoacoustic imaging, these technologies are inimitable when deployed in conjunction with PDT.

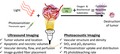

## Introduction

About 1 in 6 human deaths are cancer‐related, making it the second leading cause of death globally. The World Health Organization reported an estimated 9.6 million deaths due to cancer in 2018 alone 1. For decades, the surgical excision of tumors has been the mainstay of treatment, paired with or subsequently followed up with radiation and chemotherapy, both of which have severe side effects. A dedicated effort is underway to develop effective therapies that can be spatially and temporally localized to a tumor, with minimal damage to surrounding healthy tissue and low systemic toxicity. It would also be most advantageous for these novel therapies to possess minimal or nonoverlapping toxicity profiles when combined with other modes of therapy. Photodynamic therapy (PDT) is one such photochemistry‐based modality that imparts preferential light‐mediated cytotoxicity to target tissues while sparing surrounding healthy tissue, and is a technique that has shown tremendous potential for impacting and improving outcomes in cancer therapies 2.

**Figure 1:**
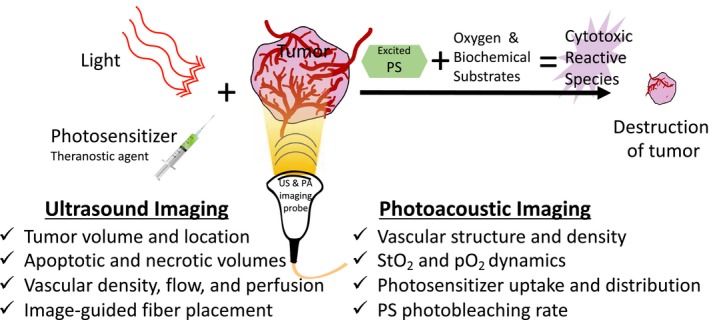
Schematic representation of PDT mechanism and list of ultrasound (US) and photoacoustic imaging surrogate markers obtained pre‐, during and post‐therapy. The photosensitizer (PS) is a phototoxic theranostic agent that upon light activation transitions into an excited triplet state and reacts with the surrounding environment (such as ground state molecular oxygen) to generate cytotoxic reactive species (such as singlet oxygen) leading to cell death. The bottom panel lists various structural and functional information that can be obtained with US and photoacoustic imaging to guide, monitor and assess PDT response. StO_2_, blood oxygen saturation. pO_2_, partial pressure of oxygen.

PDT imparts cytotoxicity via the generation of reactive species by a photosensitizer (PS) molecule irradiated by a particular wavelength of light (Fig. 1). Specifically, the optically excited PS molecule in its triplet state interacts with either molecular oxygen (type‐II PDT) in its vicinity to generate cytotoxic reactive oxygen species (ROS) or a microenvironmental substrate (type‐I PDT) to generate reactive molecular species. The PDT field has grown by leaps and bounds since its first report by Raab *et al*. in 1990 3, including the notable development of effective PSs with optical absorption maxima in the near‐infrared (NIR) range (~600–800 nm). Greater understanding of cellular mechanisms of action and effects on immune response due to PDT have also been extensively studied 4. The field has burgeoned further due to two notable advantages of PDT for cancer therapy: (1) its spatial and temporal selectivity 5 and (2) its effectiveness on chemo‐ and drug‐resistant cells 6, 7, 8. Preclinical studies have shown that PDT destroys tumor stroma and increases tumoral drug perfusion, making it an ideal complement to potent drugs that are otherwise unable to perfuse through stromal layers on their own 9, 10, 11. PDT has received regulatory approval for the treatment of several types of carcinoma and noncarcinoma pathologies, such as age‐related macular degeneration (AMD). As a clinical example, PDT of pancreatic cancer increased survival time to 12.5 months from initial diagnosis, up from the typical median survival rates for this disease (6–10 months without metastases) 12, 13, 14, 15, 16, 17, 18, 19. Moreover, in preclinical prostate, glioma and pancreatic cancer models, PDT has also shown a dose‐dependent decrease in metastases 20, 21, 22. Despite its salient features and potential in various clinical studies, a key barrier for PDT success is its variability in treatment outcomes, which may be a direct result of either under‐ or overtreating of lesions. Accurate dosimetry determined from pretreatment tumor parameters, such as size, vascular density, oxygenation status, PS uptake and online or post‐therapy monitoring, will expedite the widespread adoption of PDT technology.

Estimation of the deposited PDT dose and evaluation of the subsequent therapeutic response are a complex interdependence between the energy of delivered light, PS delivery efficiency, successful tumoral PS uptake and retention, PS clearance from the lesion, and the availability of oxygen in the treatment area 5, 23, 24, 25. Direct dosimetry parameters such as PS photobleaching via fluorescence imaging and direct singlet oxygen measurement via luminescence at 1270 nm have been used previously to gauge deposited PDT dose 24, 25, 26, 27. Either of these parameters individually or in combination were thought to be sufficient indicators of PDT dosage. However, it should be noted that these measurements are sampled at discrete locations in the tumor or are obtained from surface area‐weighted fluorescence images. Research is being pursued by several groups to understand these dosimetry nuances further via the generation of 2D and 3D maps of surrogate markers such as tumor volume, vascular function and density, tumor blood oxygenation and tumor hypoxia status 5, 28. Clinical positron emission tomography (PET), computed tomography (CT) and magnetic resonance imaging (MRI), while successful in providing data on tumor volumes, are limited by their lack of resolving power of microcirculatory activity without the use of exogenous contrast agents. There is a pressing need for imaging techniques that can monitor and assess changes in these surrogate dosimetry markers at various time points during therapy without the use of exogenous contrast agents in order to bolster treatment outcomes.

Among different clinical imaging modalities, ultrasound (US) is a ubiquitously available, nonionizing, low‐cost, portable and real‐time imaging technique that leverages the ability of acoustic waves to propagate deeply through tissues and scatter back to the receiver. It has become a primary method to measure structural and volumetric changes in tumors post‐treatment. The variations in echogenicity or acoustic impedance of tumoral and healthy tissue are instrumental in demarcating their respective boundaries within an US image. Specifically, when acoustic wavefronts generated by a transducer bounce off of the target (termed backscatter) and return to a detecting transducer, a 2‐dimensional image is generated from calculations of amplitude and the transmission‐to‐detection interval of the echo. Additional technologies, such as Doppler ultrasonography, can measure blood flow based on changes in frequency of the reflected sound wave from a moving object such as blood traveling through vessels. These unique features are primarily responsible for the prolific deployment of US imaging in both preclinical and clinical research settings. Traditional US imaging can provide information on tumor shape, size and vascular density (Fig. 1); however, it does not provide information on a tumor’s oxygenation status. When US imaging systems are integrated with a nanosecond‐pulsed laser, photoacoustic (PA) images of tissues (courtesy of acoustic wave generation via thermoelastic expansion due to light absorption by a chromophore such as hemoglobin) can be obtained utilizing the same US transmit/receiver probe (transducer), which has been demonstrated by us and others 28, 29, 30, 31, 32, 33. PA imaging provides functional information by capitalizing on the wavelength‐dependent optical absorption (*μ*_abs_) profiles of the chromophores within the imaged region, which in the case of hemoglobin stems from whether it is oxygenated or deoxygenated 29, 34, 35. Both US and PA imaging utilize similar receiver electronics. Therefore, PA imaging can be transparently integrated with widely available US imaging devices 28, 35, 36, the combination of which can provide both structural and functional information at better resolutions than MRI or PET (albeit with less penetration depth, but sufficient to obtain 3D tumor data for many types of cancers) in a single system.

In this review, we showcase the current efforts in the field of US and PA imaging and discuss their role in improving PDT outcomes of cancer—from the planning stage to predicting treatment response without the use of exogenous contrast agents, to monitoring PS uptake and vascular destruction with the use of contrast agents. The PS is a theranostic molecule which can act as an optical imaging contrast agent as well as a PDT agent. Multiwavelength or spectroscopic PA imaging confers the ability to visualize a multitude of chromophores with different optical absorption properties, whether they be exogenous and/or endogenous, via spectral unmixing 37. When delivered via nanoconstructs, PSs can act as PA contrast agents due to the high optical absorption of these carrier molecules compared to the surrounding tissue, thereby making it feasible to monitor PS uptake in the tumors. Based on whether nanoparticles were utilized to deliver PSs or exogenous contrast agents were employed to obtain surrogate imaging markers for PDT efficacy, this review is specifically divided into three parts: (1) role of US imaging in PDT, (2) role of PA imaging in PDT and (3) recent advances in multifunctional theranostic nanomaterials that can strengthen the photodynamic effect, enhance US and PA imaging contrast, and improve the precision of PS delivery to tumors, sometimes combinatorially.

## Role of US Imaging in PDT

### Monitoring tumor structural and morphological changes due to PDT

**Figure 2:**
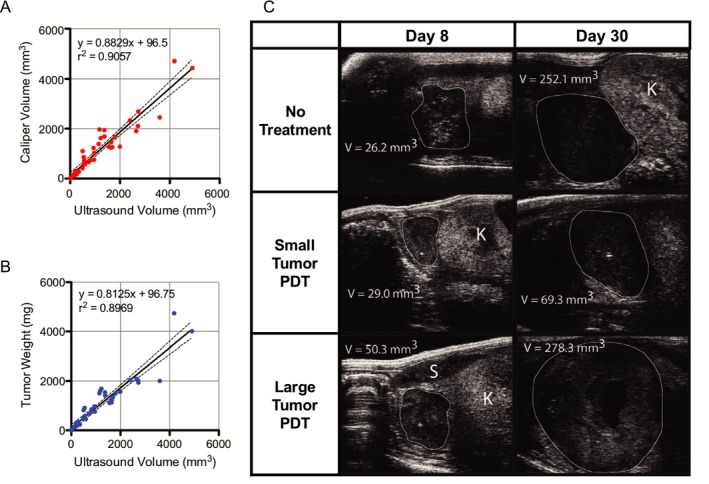
Ultrasound (US) imaging of tumor volume in vivo and correlation with ex vivo tumor weight and volume measured with calipers. (A) Plot of US‐calculated tumor volume against caliper‐measured volume and (B) weight in milligrams. High predictability of both tumor weight and caliper volume is indicated by the large coefficient of determination, indicative of the effectiveness and accuracy of using US imaging as a means of extracting tumor parameters. (C) Transverse US images of orthotopic pancreatic tumors in mouse, from which orthogonal length measurements were made, and thus volume calculated. Tumor margins and neighboring organs are differentiated based on their respective US echogenicity signatures. White outline = tumor, K = kidney, S = spleen. Adapted with permission from 38.

Ultrasound is an established and cost‐effective clinical method for identifying tumors and assessing disease progression via monitoring changes in tumor volume. Generally, cancerous tissues have different echogenicity when compared to healthy surrounding tissue structures, making their detection facile. Calculating the distance between two reflective boundaries is a common method for acquiring measurements of tumor diameters in all three (length, width and depth) spatial dimensions. Tumor volume is calculated with an ellipsoid volume formula π/6 × l(ength) × w(idth) × d(epth); however, this method can either over‐ or underestimate native tumor volumes as tumors (clinical or preclinical) are not ellipsoid, particularly as they grow larger in size. From advances in 3D reconstruction software and computing power, US imaging now enables high‐throughput acquisition and display of 3D volumetric maps of tumors. For example, in preclinical setting, accuracy of US‐based volumetric measurements has been demonstrated by Pigula *et al*., where they measured orthotopic pancreatic tumor volume with US imaging and found strong correlations with the gold standard of tumor weight and caliper‐derived volume measurements of excised tumors (Fig. 2) 38. Furthermore, the study also revealed a tumor size‐dependent response to PS benzoporphyrin derivative‐based PDT. Small tumors (<35 mm^3^) responded to a single round of PDT, while large tumors (>35 mm^3^) showed no response to the same treatment, indicating PDT dose needs to be carefully determined based on pretreatment tumor volume (Fig. 2C). A clinical example that showcases the ability of US imaging to monitor PDT responses was presented by Moore *et al*., where normal and basal cell carcinoma (BCC) thicknesses in 181 patients were monitored immediately before and up to a year after PDT 39. The study found that the thickness of BCC tumors were reduced by ~46% (i.e. 1.3 ± 0.8 mm to 0.6 ± 0.8 mm; *P*‐value < 0.001) 4–6 weeks after the PDT regimen when compared to the pretreated tumors. Similar to the study by Pigula *et al*., Moore *et al*. also found a tumor thickness‐dependent response ratio following PDT, with thinner (diameter ≤ 1.5 mm) BCC lesions responding better to PDT (85% responded) than thicker (diameter ≤ 3 mm) lesions (75% responded). Given the high‐throughput, contrast agent‐free volumetric measurements provided by US imaging, strategic decisions on the timing of subsequent PDT or combination treatments can be designed and administered for maximal efficaciousness.

**Figure 3:**
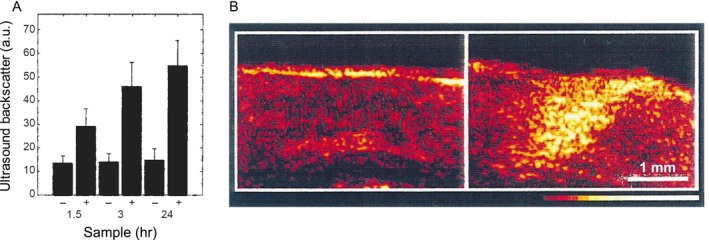
Ultrasound (US) imaging of Photofrin II‐PDT in rat brain. (A) Comparison of backscatter variations between various time points from the conclusion of treatment to imaging (40 MHz), each proceeded by a contralateral untreated tissue region serving as a control. Bars labeled “−” correspond to nontreated samples, whereas bars labeled “+” correspond to treated samples. Greatest increase in US backscatter seen 24 h after the conclusion of treatment. Error bars correspond to 1 standard deviation. (B) US imaging of control and treated contralateral regions, respectively. Tissue was imaged immediately following excision 24 h after the administration of Photofrin II‐PDT, and US backscatter intensity showed a clear uptick, indicative of a large increase in apoptotic cells in the treated region (and later confirmed histologically). Color bar range: 0 to 256. Adapted with permission from 41.

Beyond the detection of lesion boundaries and calculating tumor volume, US can also provide therapeutically relevant information on apoptotic cell death based on alterations in US backscatter intensity. Initially demonstrated by Banihashemi *et al*., the spectral slope of the US backscattered signal is related to the size of the US scatterer, in their case the nucleus. As cells undergo apoptosis, the nucleus coalesces and condenses to a point of no return (termed pyknosis), and the cell membrane undergoes stochastic bulging and deformation (termed blebbing) which thusly affects the backscatter profiles of US waves that encounter it. Currently, invasive and time‐consuming standard microscopy methods are utilized to evaluate cellular apoptosis. Given the real‐time imaging capabilities of US imaging and its sensitivity to changes in nuclear size dynamics, quantitative US imaging techniques have been successfully used to monitor apoptosis in tumors due to PDT. Banihashemi *et al*. illustrated the monitoring of cell death following PDT in SCID mice harboring human melanoma HTB‐67‐induced tumors using a broadband and high‐frequency US transducer (26 and 40 MHz central frequency), and showed a time‐dependent increase in US backscatter which corresponded to tumor cell death during Photofrin‐based PDT (110 J cm^−2^ light administered for 30 s) of subcutaneous tumors 40. Forty‐eight hours post‐PDT, they observed a decrease in US backscatter, which they attributed to the degradation of tumor nuclei. Multiple parameters derived from the spectroscopic analysis of the US backscatter frequency content were shown to correlate with tumor response early in the course of treatment by Czarnota *et al*. 40, 41. From their work, an excellent visualization of variations in backscatter as a proxy for apoptosis can be seen in Fig. 3B. Based on these observations, Banihashemi *et al*. concluded that it provides a foundation for future investigations regarding the use of spectroscopic US imaging to monitor treatment and aid in the customization of treatments, particularly PDT. With availability of high‐frequency US systems on catheters and endoscopic probes, spectroscopic US analysis could soon find its place to monitor PDT outcome for deeply situated tumors in both preclinical and clinical settings.

Acoustic frequency‐dependent attenuation in tissues limits the applicability of US imaging to a few tens of centimeters. Moreover, an inverse relationship exists between penetration depth and central frequency of the transducer (thereby the spatial resolution). The higher the transducer frequency, the greater the spatial resolution, and the shallower the penetration depth. Higher frequency transducers (>20 MHz central frequency) are primarily used in preclinical settings to monitor tumor volume in small animal models such as the rodent models and are very limitedly utilized in clinical settings for ophthalmic or skin applications. In preclinical xenograft models, Ayers *et al*. have demonstrated that the volume of tumors measured via US is more accurate and requires ~30% fewer animals to reach statistical significance when compared to standard caliper measurements 42. In another study, Ramaswamy *et al*. demonstrated that low‐field magnetic resonance imaging (MRI) and US imaging have similar accuracies in determining tumor volume and growth 43. Given the low‐cost and high‐throughput platforms that exist for 3D structural mapping, US imaging is primarily preferred for measuring most solid tumors aside from those of the brain or bone. It should be noted that the tumors in the PDT studies cited here were either subcutaneous or superficial. While US has been extensively used to evaluate the volume of tumors in the breast, pancreas, prostate, etc., and in some cases demonstrating higher accuracy than MRI, there is a dearth of clinical studies where US imaging was the primary modality to monitor PDT response of deep tissue tumors. It is only recently with the advent of various light delivery systems that studies involving PDT of tumors situated deeper within the body are being performed. We anticipate that US imaging will play a major role in monitoring tumor volumes pre‐ and post‐PDT and aid in the positioning of light delivery systems, as will be discussed in a subsequent section.

### Monitoring changes in tumor vasculature post‐PDT

**Figure 4:**
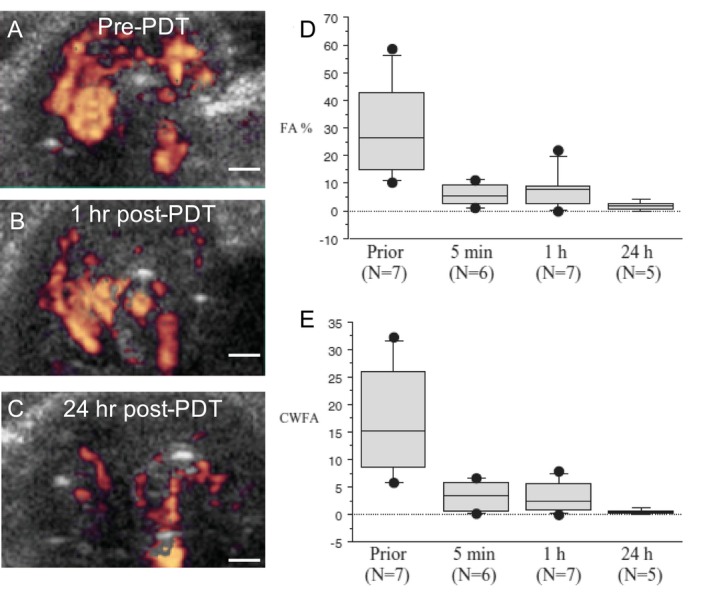
Verification of PDT efficacy using power Doppler US imaging. (A–C) Feline cutaneous squamous cell carcinoma imaged prior to Fospeg/Foscan‐PDT, 1 and 24 h post‐PDT at 652 nm. (D–E) Vascularity (FA) and blood volume (CWFA) calculated computationally from pixel analysis of power Doppler US signatures of five tumors. Scale bar = 1 cm. A reduction in FA from 29.9% to 7.8% and in CWFA from 17.1% to 3.8% occurred from therapy onset to 24 h afterward. Slight increase in vascularity at the 1‐h post‐PDT mark was thought to be attributable to partial blood vessel relaxation prior to full closure. Adapted with permission from 51.

Blood vessels are a crucial provider of nutrients, signaling highways and avenues for tumoral metastasis 44, 45, 46, 47, 48, 49. This makes them a prime target for many therapies including PDT, as their destruction or normalization has been correlated with reductions in tumor volume 50. Imaging therapy‐induced changes in blood volume and flow have the potential to be an apt surrogate for treatment efficacy, in lieu of relying on tumor morphology alone. However, microvasculature must also be studied in order to establish a full picture of what is taking place during anticancer treatments, and for this purpose, Doppler ultrasonography has been deployed 51, 52, 53. By utilizing the Doppler effect, the frequency shift between the reflected and the initial transmitted US wave is used to determine whether objects are moving toward or away from the transducer, providing data on both the movement direction and speed of the target. This applies to every moving object in the imaging window, be it in the circulatory system or surrounding tissue. Traditional Doppler US retains data on movement speed and direction while power Doppler does away with them in favor of quantifying US signal strength alone, and can measure blood volume via the assignment of colorimetric values to the strength of the detected US signal 54. Using power Doppler US, Yu *et al*. were able to illustrate tumor perfusion variations between various regions of singular RIF tumors in mice undergoing Photofrin‐PDT. Differences in PDT‐light fluence rate contributed to this variability, as higher light dose created greater levels of hypoxia heterogeneity when compared to lower light dose (75 *vs* 25 mW cm^−2^) 52. In addition, Yu *et al*. found that hypoxic tumor bases conferred a greater level of survivability to the region, despite a general increase in maturation markers and increased vascularity when compared to the regions of the tumor more proximal to the surface of the epidermis. By using the lower irradiance of 25 mW cm^−2^, however, the overall response of the tumor to therapy was far more uniform. Their work highlights the utility of monitoring tumor vascularity, particularly to understand the complex interplay between light fluence variability as it encounters a heterogenous tissue and the difficulty in predicting tumor behavior. In the case of Ohlerth *et al*.’s work, vascularity and perfusion 24 h after PDT in cats with invasive squamous cell carcinomas were monitored using power Doppler US, providing evidence of successful treatment monitoring in nonxenograft cancers 51. Power Doppler US images were collected prior to PDT, 5 min, 1 h and 24 h following PDT using either mTHPC (Foscan) or liposomal mTHPC (Fospeg) (Fig. 4), and vascular fractional area (FA) as a metric for how much of the tumor contained vasculature, mean color level (MCL) of the power Doppler US signal as a metric for how many red blood cells are present in the sample region and color‐weighted fractional area (CWFA) which is the product of FA and MCL as a unit of vascularity were calculated. Mean FA and CWFA values of the seven imaged tumors were 29.9% and 17.1%, respectively, prior to PDT, and decreased to 7.8% and 3.8, respectively, at 24 h post‐PDT**.** Overall, the study concluded that neither age, weight and concentration of hemoglobin, nor the concentration of red blood cells (RBCs) had any effects on FA and CWFA measurements and illustrated that power Doppler US imaging is a promising tool for assessing PDT outcomes in naturally occurring cancers.

### Ultrasound (US)‐guided fiber placement for PDT of deep‐seated tumors

**Figure 5:**
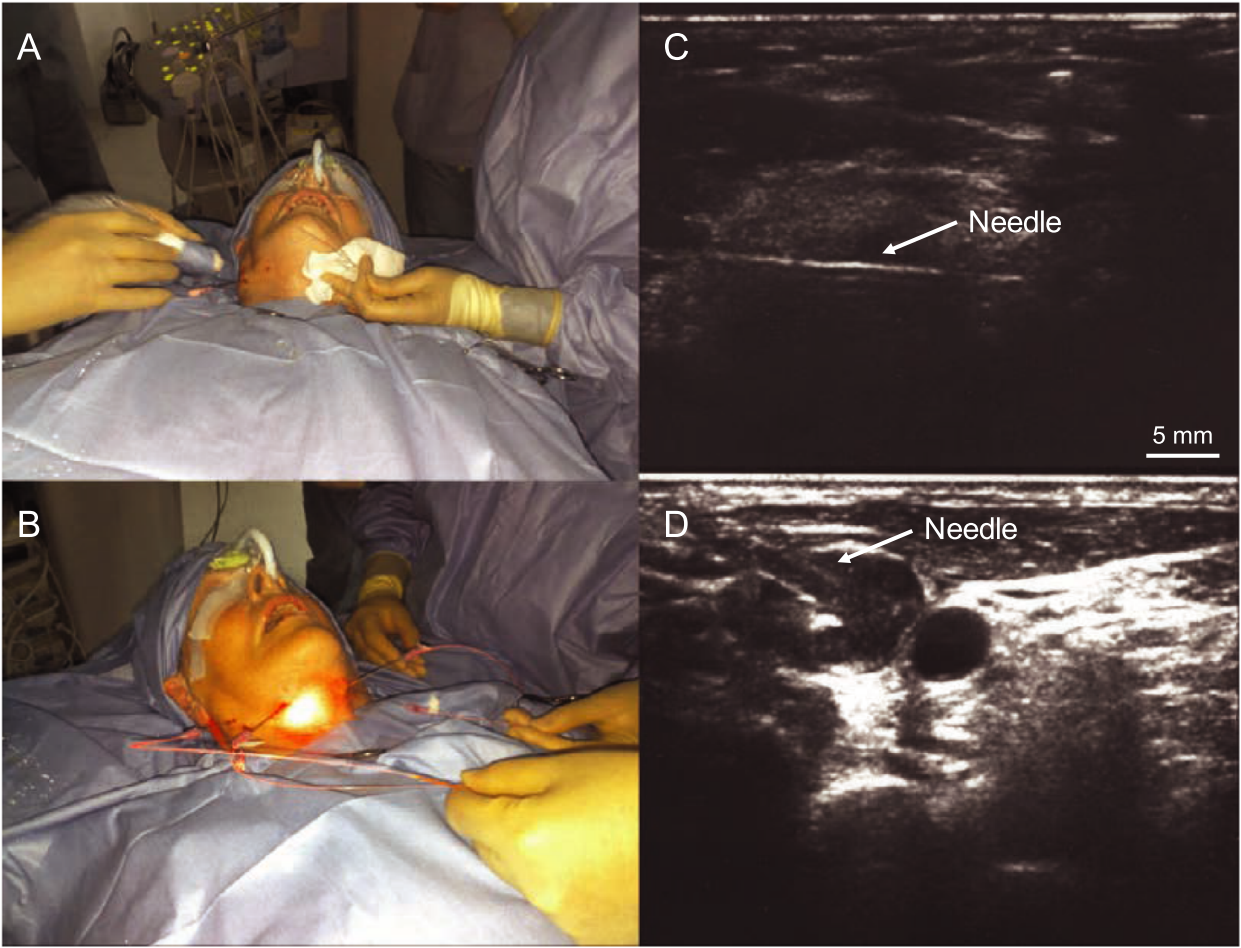
US‐guided mTHPC (Foscan)‐mediated interstitial PDT of tongue cancer. (A,B) Photographs of the patient undergoing US‐guided transcutaneous needle insertion into the tongue base and PDT to the tumor and surrounding lymph nodes. (C) US image showing needle (with light fiber tip) inserted in the tumor mass. (D) US image showing two metastatic cervical lymph nodes (black circles), into one of which the needle is being inserted. Adapted with permission from 59.

The optical absorption and scattering properties of tumors determine the light penetration depth with these tissues. Typically, light delivery to deep‐seated tumors has to be done with optical fibers via intratumor light delivery (termed interstitial PDT) 55. Tumors have heterogenous vascular content, and recently Pogue *et al*. have demonstrated that the blood content in tumors attenuates light and can affect PDT efficacy more significantly than drug distribution 56. Hence, the fiber placement in deeply seated tumors has to be carefully designed based on tumor shape, size, blood content, oxygenation and PS distribution. In order to precisely deliver light to deep‐seated (several centimeters) lesions, the use of fiber optic needles steered via US imaging has been applied in the clinic. Needles fitted with these fibers provide high contrast in US images (Fig. 5), thereby the user can position them at the desired location while steering clear of critical structures such as arteries, in order to deliver light to the target areas. Harris *et al*. utilized real‐time US‐guided optical fiber placement in mice and rabbit tumor models where a 0.5‐mm optical fiber with a cylindrical diffuser end was fitted through a 21‐gauge endobronchial US needle to deliver therapeutic light 57. Furthermore, they also demonstrated that US imaging is useful in providing anatomical information to simulate light propagation in the tumors to enable real‐time planning and dosimetry of PDT. In another study, Jerjes *et al*. have reported the successful application of the US‐guided fiber placement in the clinic using PS mTHPC (Foscan) on a wide array of maladies ranging from carcinomas, sarcomas, and hamartomas of the head, neck, and upper limbs 58. Most excitingly, they have also demonstrated its efficacy in the palliation of symptoms for several patients with stage IV carcinomas of the tongue, two‐thirds of which had been offered no further therapies (Fig. 5) 59. The majority of these patients reported improvements in speech, swallowing and breathing, and many showed tumor growth abatement at the 36‐month mark following PDT. More recently, DeWitt *et al*. utilized endoscopic US (EUS)‐guided PDT to treat locally advanced pancreatic cancer patients. Specifically, EUS was used to guide the insertion of a 1‐cm light diffuser inside the pancreatic tumor. Six out of twelve patients showed increased tumor necrosis post‐PDT 60. As PDT continues to proliferate in clinical application 2, fiber optics and US guidance will undoubtedly play a major role in improving its therapeutic effectiveness. An exciting advancement for the treatment of deep lesions has been developed by Bansal *et al*., as they have crafted implantable sources of PDT‐generating light that can be remotely activated in order to treat deep‐seated lesions 61. Once placed within the body, these devices can be activated a multitude of times, necessitating fewer surgeries when compared to the above‐mentioned fiber optic needle approach. Bansal *et al*. reported the successful attenuation of tumor growth in mice and state that their system can be used to treat tumors at a depth greater than 3 cm. In addition, these devices can be placed in 3D spatial arrangements around the tumor tissue, which could enable greater control of spatially specific light delivery and fluence adjustments to modify PDT treatments of regions of tumor response variability.

## Role of Photoacoustic Imaging in PDT

### Photoacoustic monitoring of PS uptake in the tumor

A dye molecule when excited by a specific wavelength of light releases energy in a radiative (e.g. fluorescence) or nonradiative (e.g. heat) manner. Generally, the excited electrons in the PS molecules relax to a long‐lived triplet state, which then reacts with the substrate environment to generate reactive molecular species for photodynamic therapeutic action. Some PSs also exhibit strong fluorescence, wherein the electron in the excited state can relax back to its ground state by emitting a long‐wavelength photon. In the case of nonradiative relaxation of the excited electron, heat production and in some cases the subsequent generation of PA waves can occur when irradiated with nanosecond‐pulsed laser light of a proper wavelength 62. Therefore, a PS with low fluorescence quantum yield can generally be expected to act as a good PA contrast agent 63. This is clearly demonstrated by Ho *et al*., where five different PS molecules were evaluated for their fluorescence and PA properties 30. Zinc phthalocyanine with ~37.5% less fluorescence quantum yield showed a 50% higher PA quantum yield than other commonly used PSs such as protoporphyrin IX 30. Furthermore, the optical absorption coefficient of a PS molecule in “free” form might not be sufficient to obtain a sufficient signal‐to‐noise ratio (SNR) for PA imaging *in vivo*. As PA signal generation is primarily dependent on the optical absorption properties of the object being imaged, PS molecules encapsulated within nanosystems can provide enhanced PA contrast via the quenching of fluorescence of the PS molecules that are in close proximity to each other 64. For example, Lovell *et al*. showed that PS‐conjugated lipids can be used to fabricate nanovesicles called porphysomes that provide high PA contrast and can be used as image‐guided theranostic agents 65. When encapsulated in a porphysome, the PA intensity of MB increased by a factor of 5 within the 750–800 nm excitation range. Several studies are ongoing to monitor PA signal change as these nanoliposomal constructs or PS molecules degrade in response to PDT or the tumor microenvironment. Verifying the presence or absence of PS in the lesion is a key deciding factor for the continuation or cessation of further light irradiation for PDT. A more detailed discussion on nanotheranostic agents is provided in a later part of this review.

### Photoacoustic monitoring of PDT‐induced vascular damage

**Figure 6:**
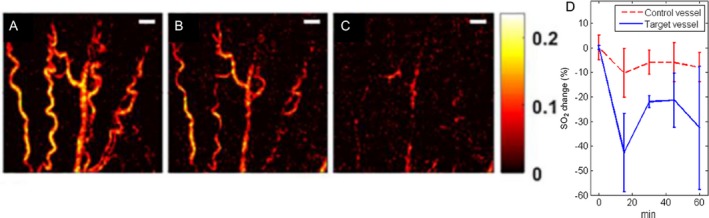
Monitoring changes in vasculature due to PDT using PA imaging. Validation of pre‐ and post‐HPPH‐PDT changes in vasculature of basal cell carcinoma‐harboring mice with PA imaging, taken before PDT administration (A), 1 min post‐PDT (B) and 10 min post‐PDT (C). Vascular area was reduced by 49% and 90%, and blood vessel diameter was reduced 23% and 63%, respectively. Scale bar = 500 μm. (D) Example of StO_2_ decrease and return to near‐endogenous levels during verteporfin‐based PDT in the mouse ear blood vessel. Adapted with permission from 66 and 71.

As previously mentioned, vasculature provides a singular source of a multitude of tumor‐related targets. US imaging has been proven to be a powerful tool for assessing changes in blood flow, cellular morphology, and general tumor volume and structure during PDT. PA imaging further adds to the diagnostic arsenal by providing a means to image vascular structural changes, by imaging the endogenous chromophore hemoglobin in blood. The optical absorption properties of hemoglobin are distinct and higher than normal tissue, which is typically highly optically scattering. This variation of optical absorption properties enables PA imaging to generate maps of vascular structure from deep tissues. The work by Rohrbach *et al* and Xiang *et al*. beautifully illustrates the utility of monitoring vascular destruction due to PDT 66, 67. Rohrbach *et al*. utilized PA imaging to show that total vascular area was reduced by 90% following 10 min of PDT and average blood vessel diameter was reduced by 63% (Fig. 6A–C). Similarly, Xiang *et al*. demonstrated that PA imaging can aid in real‐time determination of vascular size, structure and dose‐dependent damage in mice with basal cell carcinoma following 2‐(1‐hexyloxyethyl)‐2‐devinyl pyropheophorbide‐a (HPPH)‐based PDT. Specifically, a 20% drop in blood vessel size at the 5‐min post‐PDT mark and a 60% size reduction by 20 min post‐PDT (4 mJ cm^−2^ light dose) in a 140‐μm blood vessel were observed. Several other optical imaging technologies such as laser Doppler imaging, laser speckle imaging, intravital microscopy and optical frequency domain imaging have been utilized to monitor vascular damage post‐PDT 68. Though resolution is sacrificed to obtain vascular information at deeper depths in PA imaging, the previously mentioned optical imaging modalities cannot simultaneously obtain vascular structure, vascular function and PS accumulation profiles in the tumor at deeper depths. Furthermore, the nanosecond‐pulsed laser utilized for PA imaging can also be used for PDT activation such as that demonstrated by Xiang *et al*, where vascular PDT with PS protoporphyrin IX (PpIX) was performed with a 532‐nm nanosecond‐pulsed laser 67. Overall, these studies point to the utility and potential of PA imaging to optimize PDT by providing real‐time feedback on dose‐dependent vascular changes at deeper depths within tumors.

### Photoacoustic monitoring of PDT‐induced oxygen saturation change and predicting therapeutic efficacy

**Figure 7:**
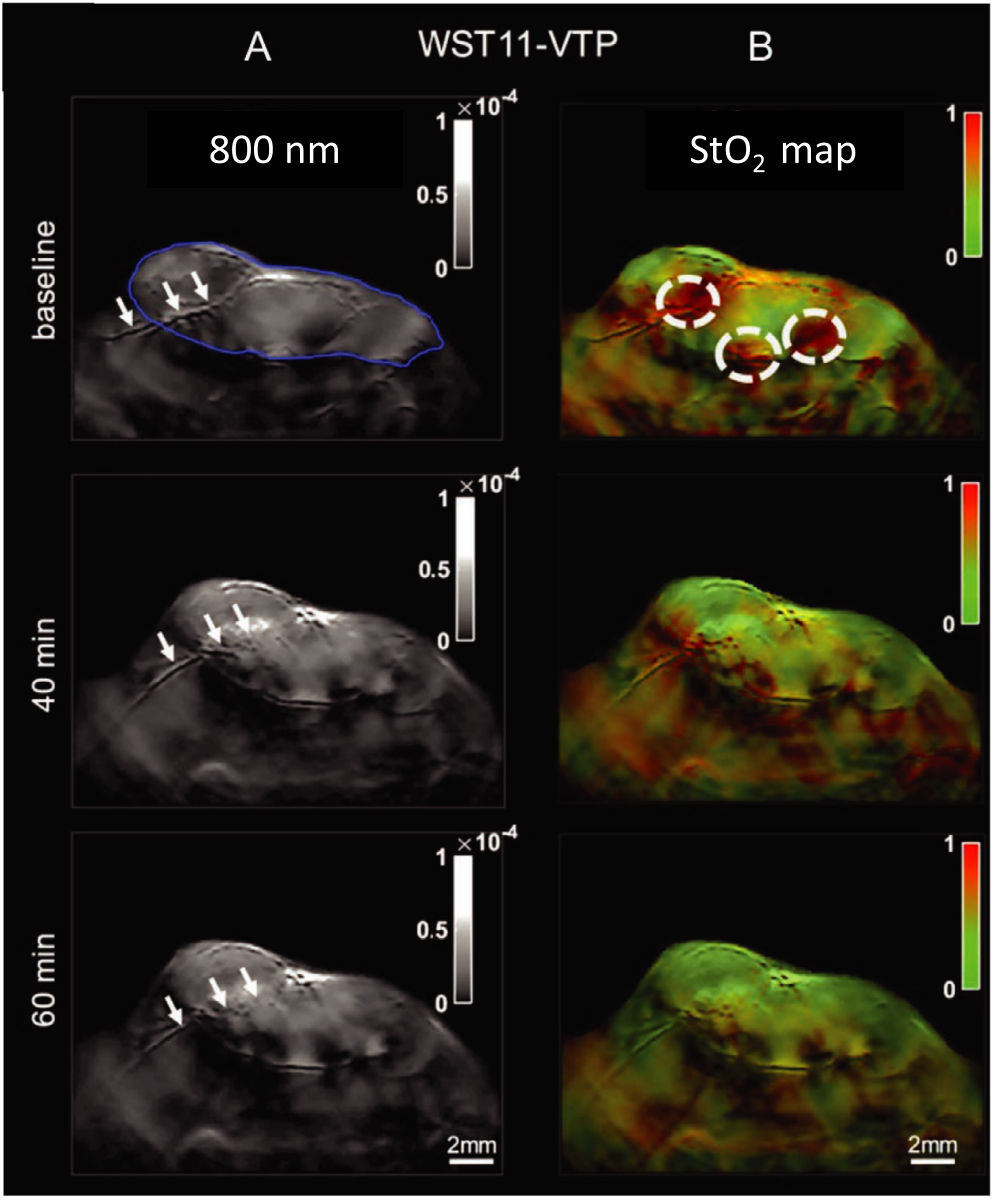
Photoacoustic imaging (multispectral optoacoustic imaging) of StO_2_ levels in renal carcinoma tumors following WST‐11‐mediated PDT. (A) Imaging of vascular morphological changes at baseline, 40 and 60 min post‐PDT. Blue outline of top‐left panel indicates tumor boundary, and white arrows point to a major vessel traversing through the tumor. At 60 min post‐PDT, clear structural ablation of this vessel can be seen. (B) StO_2_ map of treated region, indicating time‐dependent drop in StO_2_ content (shift from red (high) to green (low) StO_2_ values). Adapted with permission from 72.

Hemoglobin in its oxygenated or deoxygenated state has distinguishable optical absorption properties, that is distinct spectra. Based on these differences, PA signals obtained at multiple wavelengths can be unmixed to quantify blood oxygen saturation and to understand the contribution of oxygenated and deoxygenated hemoglobin in a particular image voxel 69. Studies probing StO_2_ with other optical spectroscopic imaging techniques demonstrated that tumors with low StO_2_ or hypoxic pretreatment values did not respond to PDT 70. While these studies have provided insights into PDT’s mechanism of action and impact on tumor vascular function, PA imaging has a major advantage over other spectroscopic methods as it allows noninvasive, longitudinal and highly spatially resolved monitoring of StO_2_ content of the whole tumor volume. Shao *et al*. demonstrated the capabilities of PA imaging to monitor real‐time changes in oxygen levels post‐PDT while simultaneously measuring alterations in the blood vessel size 71. In their study, a 40% drop in StO_2_ levels ~18 min post‐PDT followed by a recovery to 20% less than pretreatment values was observed, while constriction of the blood vessels was observed 30 min after PDT (Fig. 6D). Treatment‐induced time‐dependent mechanisms of vascular shutdown are poorly understood phenomena. Monitoring of vessel size with PA imaging post‐treatment, such as that demonstrated by Shao *et al*. 71 and Xiang *et al*. 67, showcases examples of the need for imaging modalities such as PA for providing deeper insights into therapeutic mechanisms of action. In another study, Neuschmelting *et al*. performed vasculature‐targeted PDT with PS WST11 (activated at 753 nm and cleared from the mouse body in 20 mins) and monitored StO_2_ levels within the tumor vasculature 72. The success of the PDT regimen’s goal of vascular destruction was verified from a 60% reduction in StO_2_ content one hour following PDT (Fig. 7). Furthermore, they also showed that PA‐pulsed illumination alone, at a wavelength of 753 nm, was sufficient to activate PS WST11. This enabled PA imaging of PS uptake, the initiation of PDT and the monitoring of the therapeutic end point of vascular shutdown, all with the same system.

**Figure 8:**
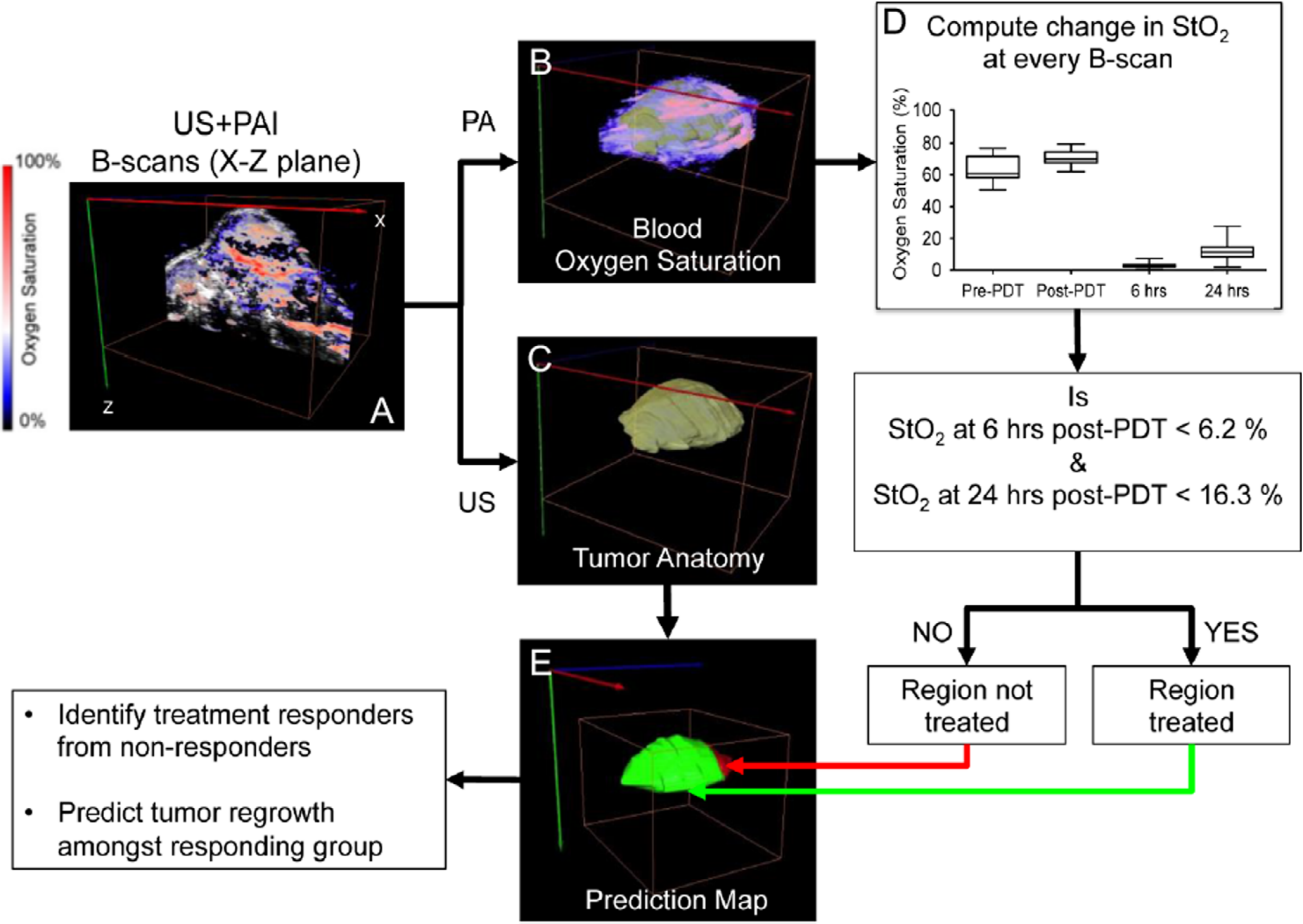
Schematic representation of the image processing workflow of predicting PDT treatment response from 3D ultrasound (US) and PA images. (A) Example slice of combined PA and US image of tumor anatomy and StO_2_ content, respectively. (B) 3D StO_2_ overlaid on tumor anatomy map, and (C) US‐imaged tumor anatomy alone. Each StO_2_ value of a given region in a slice (A) is then fed into D: the computational pipeline for determining StO_2_ variability and thus treatment responsiveness. Should the StO_2_ values satisfy the requirements of both the 6‐h and 24‐h post‐PDT cutoffs, a Boolean value corresponding to red (not responsive to treatment) or green (responsive to treatment) is then used to reconstruct a 3D map of regional tumor responsiveness (E). Adapted with permission from 73.

PA imaging’s ability to generate 3D maps of tumor oxygenation status opens up new avenues for predicting PDT treatment efficacy. Given the heterogenous microenvironment of tumors, PA imaging can be utilized to map regions within the 3D tumor volume that were responsive or nonresponsive to a treatment 73. This is not possible with other spectroscopic optical imaging techniques that can measure oxygen saturation 74. Using benzoporphyrin derivative (BPD) as their PS, Mallidi *et al*. analyzed 3D PA images of oxygen saturation and built statistical algorithms to predict recurrence of glioblastomas post‐PDT (Fig. 8). To begin with, the authors studied the oxygen saturation changes in mice at various time points post‐PDT. The study showed an 85% decrease in StO_2_ values in responsive tumors 24 h following PDT, while nonresponsive tumors did not show a significant change in StO_2_ values at 6 h or 24 h post‐PDT. Another important factor that can affect PDT response is the proper interval between PS administration and the initiation of PS excitation, termed the drug–light interval (DLI). Mallidi *et al*.’s study corroborates previous findings in the field that BPD‐based PDT is most effective when the PS is localized to the vasculature (shorter DLI) than within the tumor cells themselves (longer DLI). Utilizing 3D StO_2_ maps at 24 h post‐PDT available via PA imaging, Mallidi *et al*. built a prediction model that could precisely gauge areas of nonresponsiveness within a given tumor. Recurrence that could visually be monitored only several weeks post‐PDT can now be evaluated via PA imaging within 24 h post‐PDT. This methodology provides a powerful tool that can be utilized in designing subsequent PDT doses or in designing combination treatments with other anti‐angiogenic therapies 73 (Fig. 8).

### Photoacoustic measurement of oxygen content in tumors

**Figure 9:**
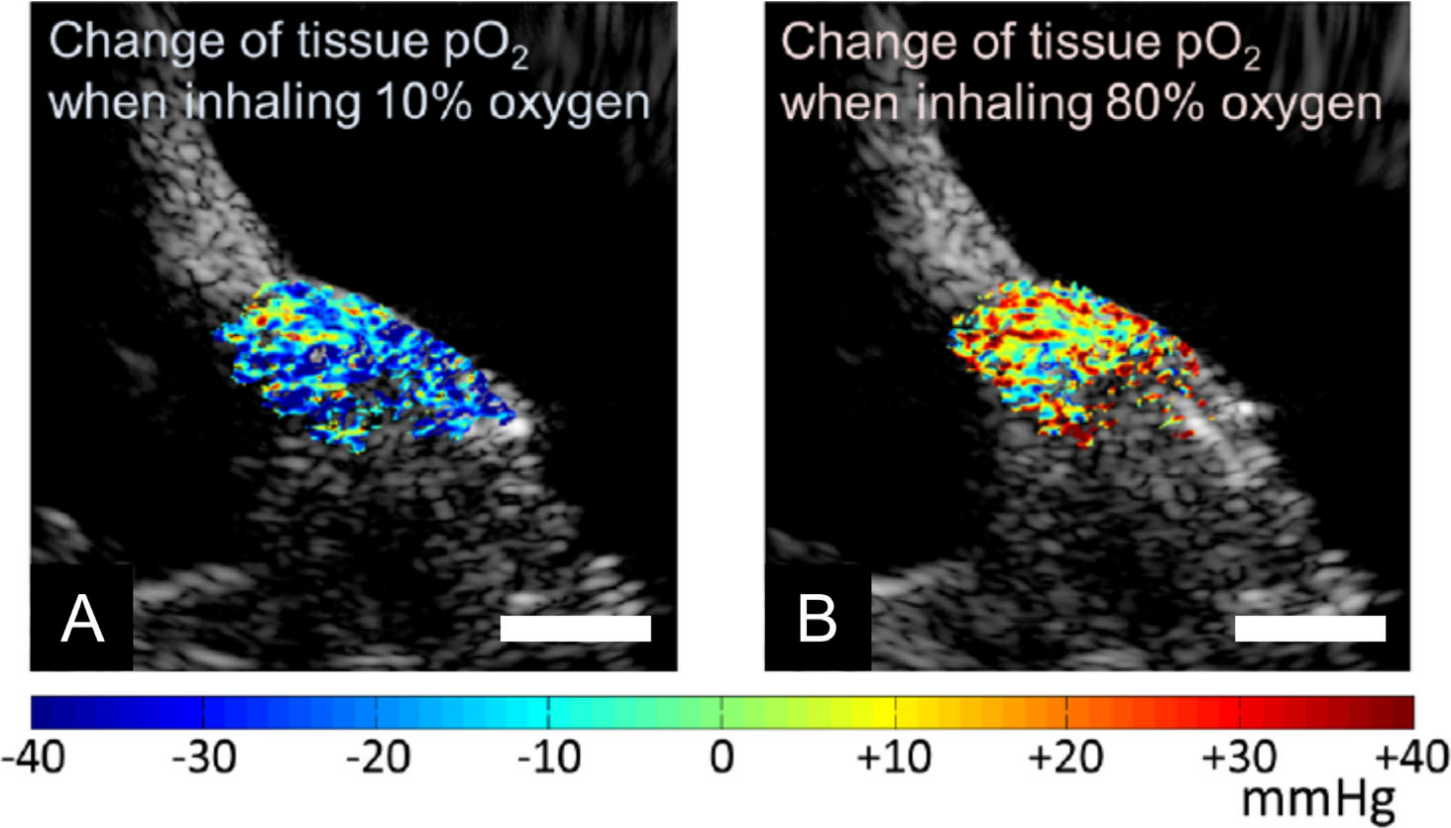
Demonstration of photoacoustic lifetime oxygen monitoring in vivo. Ultrasound images (grayscale) overlaid with pseudocolored PALI images of various oxygen concentrations. Both the left and right images are from the mouse hind limb. The localized partial pressures of oxygen of a resting mouse inhaling either 10% (A) or 80% (B) oxygen, respectively. Scale bar = 5 mm. Adapted with permission from 76.

The presence of oxygen in the vicinity of an excited PS molecule is key for the generation of reactive oxygen species. However, photochemical depletion of local oxygen content is generally not accounted for in the same fashion as is the presence of PS in the lesion due to limited availability of imaging techniques that can measure partial pressure of oxygen pO_2_ values. Several studies monitored PDT’s impact on dynamic changes in tissue oxygenation (pO_2_ levels) with invasive single micro‐electrodes, such as the Eppendorf probes. For example, Pogue *et al*. demonstrated that pO_2_ changes were heterogenous and that the lower pO_2_ regions responded differently than the higher pO_2_ regions, potentially as a factor of lesser blood flow rates 75. The Eppendorf probe‐based methods to measure pO_2_ have significant limitations, including their inability to produce 2D tissue pO_2_ maps, preventing them from being widely used in PDT research. Recently, Shao *et al*. have reported a direct and noninvasive PA lifetime imaging method that can produce 2D maps of tissue pO_2_ (Fig. 9). Here, the excited triplet state of oxygen‐sensitive dye methylene blue (MB) was used to assess the oxygen content within cells 76. By “pumping” the MB molecule with a 650‐nm laser and causing it to shift into its excited triplet state, then “probing” (matching) its emission wavelength with an 810‐nm laser, the resulting PA signal may be teased out with an US transducer. This technique, called photoacoustic lifetime imaging (PALI), could become a powerful tool for the rapid assessment of PDT with high spatial resolution of cellular oxygen content. Data on the localization of oxygen in tumors as a factor of pH and temperature alterations from therapy or inflammatory responses would be extremely useful for understanding the more nuanced behaviors of cells as they undergo PDT.

## Theranostic Nanomaterials for Image‐Guided PDT

The effectiveness of a PDT regimen depends on PS deliverability to the tumor, confirming the intratumoral or intracellular localization of the PS delivery, and utilizing the proper wavelength and light fluence to initiate PDT. A major concern for the older generation of PSs had been off‐site cutaneous photosensitivity which decreased the enthusiasm for this modality 77. The recent burgeoning of the nanotechnology field has enabled the encapsulation or absorption of PS molecules into nanoconstructs which can target tumors passively, through leaky vasculature and enhanced permeability and retention 78, or actively via various mechanisms as reviewed elsewhere 79, 80. The nanotechnology‐based specific delivery of the PS has dramatically decreased off‐site toxicity, and their combination with imaging modalities that can monitor the distribution and activity of these nanoconstructs has renewed interest in image‐guided PDT 77, 81, 82, 83, 84. All manner of therapeutic agents, from tumor‐specific antigens on the outer surface to ribonucleic acids contained within a nanoparticle, can be combined and tuned to specific signatures such as pH, cell surface markers, or the transcriptional profiles of the target tumor. In addition, contrast agents may be placed on the periphery or within the core of a nanoparticle, as can various sources of ROS generation. Overall, the therapeutic nanotoolbox has greatly increased in size and scope, to include the delivery of multiple PSs and/or other therapeutics. From these advances, understanding each one of them in the context of biocompatibility, tumoral destruction and synergistic or discordant effects is essential for their proper deployment. Specifically, in this section we will review the nanoparticles that can provide either US or PA contrast enhancement and/or ROS generation while simultaneously delivering PSs to the tumor with increased precision.

Gas‐filled microbubbles are excellent US contrast agents as gaseous media has greater acoustic impedance than that of fluids and biological tissues. Due to their size, they are purely intravascular tracers and do not extravasate into the tumor interstitium. The US signal intensity is dependent on the microbubble (either passively or specifically targeted to a cancer biomarker) concentration at the target site. Continuous imaging of microbubbles in the tumor vasculature can be quantified, color coded and displayed on top of structural US images to identify regions of abnormal perfusion 85. Cornleis *et al*. showed that contrast‐enhanced US (CEUS) imaging can be used to image PS WST11‐based PDT‐induced vascular damage 86. Though survival and treatment efficacy were not assessed in this study, CEUS images showed good correlation with histology images of necrosis. Microbubbles can also act as PS delivery agents, wherein PSs can be encapsulated in the core, absorbed onto the surface or incorporated into the lipid layers of the microbubble as demonstrated by Huynh *et al*. 87. Furthermore, the outer shell of the microbubble can be coated with proteins, lipids and polymers for enhanced tumor targeting 88. Unfortunately, microbubble deployments have been limited by low stability, short half‐lives and a lack of prolonged circulation time, all of which complicate the assessment of successful PS delivery. The work of Park *et al*. has sought to address these shortcomings by developing a pH‐dependent microbubble that releases CO_2_ at tumor‐specific pH, which greatly enhanced US signal for the guidance of subsequent PDT via the PS chlorin e6 payload contained within 89. Similarly, You *et al*. developed a novel porphyrin‐grafted lipid microbubble in which the specificity of the PS payload was assisted by US‐targeted destruction of the microbubbles, adding further spatial control and improving its therapeutic efficacy 88. Another exciting development in the realm of enhanced US‐guided PDT came from the research of Sun *et al*., in which they combined US‐targeted PDT with gene therapy to treat triple‐negative breast cancer. They achieved this goal by using cationic porphyrin lipid microbubbles loaded with HIF1alpha‐siRNA, providing a therapeutic with both enhanced contrast and the added element of interfering with tumor function on the level of the transcriptome 90. The complexities of gene expression provide a multitude of therapeutic targets, from transcription factors (TF) 91 and miRNA‐mediated transcriptional up‐ or downregulation 92, to cis‐ and transchromosomal long noncoding RNA (lncRNA) bridges, 93, 94, 95, all of which contribute to the proteomic profiles and organellar architecture of a cell 96. In the case of Sun *et al*.’s approach, targeting the transcripts of what would then be translated into the HIF1alpha TF provided a means of attacking the source of several downstream products of unbridled cellular activity, as HIF1alpha’s transcriptional influence has been correlated with the increase of tumoral glycolysis and vascular endothelial growth factor (VEGF) translation, both of which are hallmarks of tumor proliferation 97 and important factors of PDT's mechanism of action on tumors 5.

Another arena of nanoparticle‐based PDT agents are organic and inorganic nanomaterials, alone or in combination with PSs, which can generate ROS 98. As an added bonus, metallic plasmonic nanoparticles are known to exhibit several fold higher absorption spectra than endogenous tissue chromophores, making them conducive to PA monitoring of their uptake in tumors. For example, Lin *et al*. reported the development of a high‐PS capacity gold nanoparticle housing PS Ce6 (GV‐Ce6), which allows for 3 modes of imaging (near‐infrared fluorescence, thermal and PA imaging) in addition to improved tumoral uptake of the PS payload from heat‐induced release of the Ce6 cargo 99. Photothermal and photodynamic activation of their gold vesicle‐Ce6 NP takes place at the same wavelength of 671 nm, as does the three modes of imaging. Lin *et al*. verified the tumor killing ability of their NP while also illustrating the spatially specific heating caused by 731 nm illumination in the tumors. Pronounced decrease in tumor volume was observed from activation of the GV‐Ce6 NP in addition to tumoral pyknosis. In another study, Lin *et al*. reported the synthesis of two‐dimensional tellurium nanosheets which are capable of producing ROS and show high PA imaging performance due to their strong near‐infrared absorbance, which confers greater activation and imaging depth 100. In addition, Lin *et al*. suggest that their system can be engineered as a nanoplatform for simultaneous PA imaging and PDT. In another study, Ding *et al*. described the preparation of nanocrystallites composed of the water‐insoluble PS zinc(II)‐phthalocyanine in the form of nanodots, created by applying a cryodesiccation‐driven crystallization approach 101. Modification of the surface of the nanodots with Pluronic F127 and folic acid endowed them with excellent water solubility and stealth properties in blood, which lengthens their circulation time by avoiding immunological detection and destruction. Under NIR excitation at 808 nm, the nanodots are shown to produce singlet oxygen and are of low cytotoxicity. Hou *et al*. reported the synthesis of Cu‐Sb‐S NPs paired with poly(vinylpyrrolidone) (PVP), which exhibited higher photothermal efficiency and thus PDT effectiveness in addition to improved contrast over prior copper‐based NPs 102. Sun *et al*. synthesized a perylene diimide zwitterionic polymer PDS‐PDI via atom transfer radical polymerization (ATRP), which is capable of inducing both PDT and photothermal therapy (PTT) 103. Finally, Kim *et al*. reported the production of a pluronic nanogel‐based carrier for PSs chlorin e6 and gold nanorods, functionalized with chitosan, for a reversal of previously reported PTT‐PDT dual therapy sequence 104. Controlling the quenching of a PS during dual‐phototherapeutics involving gold nanorod‐mediated PTT had been elusive prior to their novel nanogel design, generally allowing only for the deployment of PDT after PTT. Kim *et al*.’s nanogel allowed for the spatial separation of the gold nanorods and chlorin e6 PS, and thus no PTT‐mediated quenching of chlorin e6 took place. As such, Kim *et al*. were able to deploy PDT prior to PTT, resulting in marked tumor size regression in SCC7 tumor‐bearing mice, followed by tumor elimination without return at 10 days post‐therapy onward. The field of gel‐based PDT and PTT agents is an especially exciting one, as future applications may provide greater insight into sensitizer‐tumor localization (imaged with US and PA techniques) following treatment courtesy of novel isotropic sample expansion techniques 105. Further examples of theranostic nanoparticles for PDT are listed in Table 1 and are illustrative of the diversity of these techniques and their efficacious potential in treating a wide range of cancers.

**Table 1:** List of theranostic nanomaterials that deliver photosensitizer and also act as US and/or photoacoustic contrast agents

Nanomaterial	Model	Photosensitizer dose/DLI	Imaging parameters	Tumor targeting	Ref
Nanoagents for US contrast and delivering PS					
Porphyrin‐grafted lipid microbubble (PGL‐MB)	PC3 Human prostate cancer xenograft in mice	Porphyrin 650 nm, 200 mW.cm^−2^ 4 h after low‐ frequency US (LFUS)	US @ 1 MHz to monitor NP accumulation at tumor	Porphyrin‐tumor affinity, US‐targeted microbubble destruction (UTMD)	88
Ce6‐loaded CaCO_3_ core and PEG shell	MCF‐7 Human breast cancer *in vitro*	Ce6 671 nm, 6 J.cm^−2^ 0 h DLI	US @ 40 MHz to assess NP’s US contrast for 3 h	CaCO3 causing PS release at tumoral pH	89
Porphyrin‐grafted lipid (CPGL) microbubble loaded with HIF 1α siRNA (siHIF@CpMB)	MDA‐MB‐231 Human breast cancer injected in mice	Porphyrin 650 nm, 200 mW.cm^−2^ 6 h DLI	US @ 3‐12 MHz to monitor MB uptake in tumor	Porphyrin‐tumor affinity, UTMD	90
Nanoagents for photoacoustic contrast and delivering PS					
Dox‐loaded, folate receptors α (FRα) targeted MTX‐decorated self‐assembled zinc phthalocyanine–soybean phospholipid complex NPs (DZSM)	4T1 Human breast cancer cells Subcutaneous tumors	ZnPc‐SPC (ZS) complex 638 nm, 1000 mW.cm^−2^ 24 h DLI	PA imaging @ 638 nm to monitor DZSM accumulation at tumor site	Folic acid (FA) receptor‐mediated tumor uptake	140
Hyaluronic acid (HA) coupled with chlorin e6 (Ce6) via adipic dihydrazide (ADH) forming HA‐ADH‐Ce6 conjugates and self‐assembly into HACE NPs.	A549 Human lung cancer Subcutaneous tumors	Ce6 660 nm, 160 mW.cm^−2^ 24 h DLI	PA imaging @ 680 nm to monitor HACE NP accumulation at tumor	HA specificity to CD44 on tumor	127
Ce6 bound to HA nanoparticle with perfluorohexane core (PFH@HSC)	MDA‐MB‐231 Human breast cancer Subcutaneous tumors	Ce6 660 nm, 100 mW.cm^−2^ 4 h DLI	PA imaging @ 690‐900 nm to monitor hypoxia relief and PFH@HSC accumulation at tumor	EPR and HA specificity to CD44 on tumor	128
Diketopyrrolopyrrole (DPP)–triphenylamine (TPA) NP	HTC‐116 Human colorectal cancer cells Subcutaneous tumors	DPP‐TPA 660 nm, 1 W.cm^−2^ 2 h DLI	PA imaging @ 680 nm to monitor DPP‐TPA NP accumulation at tumor	EPR and PA‐guided activation	129
Perylene diimide zwitterionic polymer (PDS‐PDI)	MDA‐MB‐231 Human breast cancer injected in mice	Perylene‐3,4,9,10‐tetracarboxylic diimide (PDS) 660 nm, 500 mW.cm^−2^ 6 h DLI	PA imaging @ 660 nm to monitor PDS‐PDI accumulation at tumor	EPR and PA‐guided activation	103
Tellurium nanosheets functionalized with glutathione (GSH)	HepG2 Human liver cancer injected into mice	Te nanosheets 670 nm, 160 mW.cm^−2^ 0 h DLI	PA imaging @ 680‐980 nm to confirm tumor uptake of Te nanosheets + GSH	EPR and PA‐guided activation	100
Cell membrane‐derived shell and a methylene blue and cisplatin (Pt) loaded gelatin nanogel core (MPV)	4T1 Murine breast cancer orthotopic tumors in mice	Methylene blue 671 nm, 450 mW.cm^−2^ 1 h and 4 h DLI	PA imaging @ 680 nm to monitor intratumoral deposition of MPV	EPR and PA‐guided activation	130
Pc core and four ethanolamine and phthalocyanine‐difunctionalized poly(glycidyl methacrylate) arms NP (Pc‐PGEA/Pc‐3) containing p53	C6 Rat glioma injected in mice	Pc‐PGEA/Pc NPs 700 nm, 800 mW.cm^−2^ 0 h DLI	PA imaging @ 680‐980 nm to monitor Pc‐PGEA/Pc accumulation at tumor	EPR and PA‐guided activation	131
BODIPY within amphiphilic DSPE‐mPEG5000	A549 Human lung cancer subcutaneously injected in mice	BODIPY 730 nm, 200 mW.cm^−2^ 0.5 h DLI	PA imaging @ 760 nm to assess enhanced permeability and retention (EPR) and lysosomal accumulation of BODIPY NP	Direct injection, BODIPY accumulation in acidic lysosomes	132
Lecithin/DSPE‐PEG‐FA outer shell containing PCM core housing DOX and diketopyrrolopyrrole (DPP)‐BT dye, functionalized with FA (P(DPP**‐**BT/DOX) NP)	HeLa Human cervical cancer subcutaneously injected into mice	DPP‐BT 730 nm, 1000 mW.cm^−2^ 24 h DLI	PA imaging @ 730 nm to monitor P(DPP‐BT/DOX) NP accumulation at tumor	FA receptor‐mediated tumor uptake	133
Silicon 2,3‐naphthalocyanine bi(trihexylsilyloxide) (SiNc)	HT‐29 Human colorectal cancer injected into mice	SiNC 770nm, 40 mJ .08 – 1 h DLI	PA imaging @ 680‐860 nm to monitor SiNC presence in tumor and assessment of SiNC PA signal strength	EPR	134
Porphyrin‐ or phthalocyanine‐bridged silsesquioxane nanoparticles (BSPOR and BSPHT)	MCF‐7 Human breast cancer cells *in vitro*	BSPOR/BSPHT 800 nm, 4300 mW.cm^−2^ 24 h DLI	PA imaging @ 700 nm to monitor BSPOR and BSPHT accumulation at tumor	Porphyrin‐tumor affinity	135
Artificial red blood cell loaded with oxygen (IARC)	MCF‐7 Human breast cancer injected into mice	ICG 808 nm**,** 100 mW.cm^−2^ 0.5 h DLI	Spectroscopic PA imaging to monitor ICG, HbO_2_ and Hb accumulation at tumor	EPR	109
ICG‐loaded PEGylated silver nanoparticle core/polyaniline shell (Ag@PANI) nanocomposites (ICG‐Ag@PANI)	HeLa Human cervical cancer subcutaneously injected into mice	ICG 808 nm/1000 mW.cm^−2^ 24 h DLI	PA imaging @ 808 nm to monitor accumulation of ICG‐Ag@PANI at tumor	EPR	136
ICG‐HA nanoparticle embedded with single‐walled carbon nanotubes ICGHANP/SWCNTs (IHANPT)	SSC7 Human oral cancer subcutaneously injected in mice	ICG 808 nm/800 mW.cm^‐2^ 24 h DLI	PA imaging @ 808 nm to monitor IHANP accumulation at tumor	EPR and IHANPT specificity to CD44 on tumor	137
MgO_2_ NP in ICG and hyaluronic acid (HA) NP	SSC7 Human oral cancer xenografted in mice	ICG 808 nm, 800 mW.cm^−2^ 6 h DLI	PA imaging @ 808 nm to monitor NP accumulation at tumor	HA specificity to CD44 on tumor	107
Hyaluronic acid (HA)–cystamine–cholesterol (HSC) self‐assembling conjugate incorporating IR780 (HSCI NPs)	MDA‐MB‐231 Human breast cancer injected in mice	IR780 808 nm, 800 mW.cm^−2^ 0 h DLI	PA imaging @ 680‐980 nm to monitor HSCI NP accumulation at tumor	EPR and HA specificity to CD44 on tumor	138
Iridium‐cyanine nanoparticle (IrCy)	4T1 Murine breast cancer syngeneic xenografts in mice	Iridium dye 808 nm, 50 mW.cm^−2^ 24 h DLI followed by IrCy readministration at 48 h and PDT at 72 h	PA imaging @ 815 nm for detecting cyanine dye to monitor biodistribution and accumulation of IrCy at tumor	EPR and PA‐guided activation	139
Cu‐Sb‐S functionalized with poly(vinylpyrrolidone) (PVP‐Cu‐Sb‐S) NP	4T1 Murine breast cancer syngeneic xenografts in mice	PVP‐Cu‐Sb‐S NP 808 nm, 1000 mW.cm^−2^ 0 h DLI	PA imaging @ 808 nm for monitoring tumor uptake of PVP‐Cu‐Sb‐S NPs	EPR and PA‐guided activation	102
Zinc(II)‐phthalocyanine nanodots, PEG‐folate/ZnPc nanodots (FA‐ZnPcNDs)	CNE‐2 Human nasopharyngeal cancer injected into mice	ZnPC 808 nm, 500 mW.cm^−2^ 2 h DLI	PA imaging @ 808 nm to monitor FA‐ZnPcND accumulation at tumor	FA receptor‐mediated tumor uptake	101
Polypyrrole with astaxanthin‐conjugated bovine serum albumin polymer (PPy@BSA‐Astx)	MBA‐MD‐231 Human breast cancer *in vitro*	Astaxanthin 808 nm, 300 mW.cm^−2^ 6 h DLI	PA @ 808 nm to monitor PPy@BSA‐Astx US signal production	Passive targeting	141

### Enhancing oxygen content in the microenvironment via nanoagents

**Figure 10:**
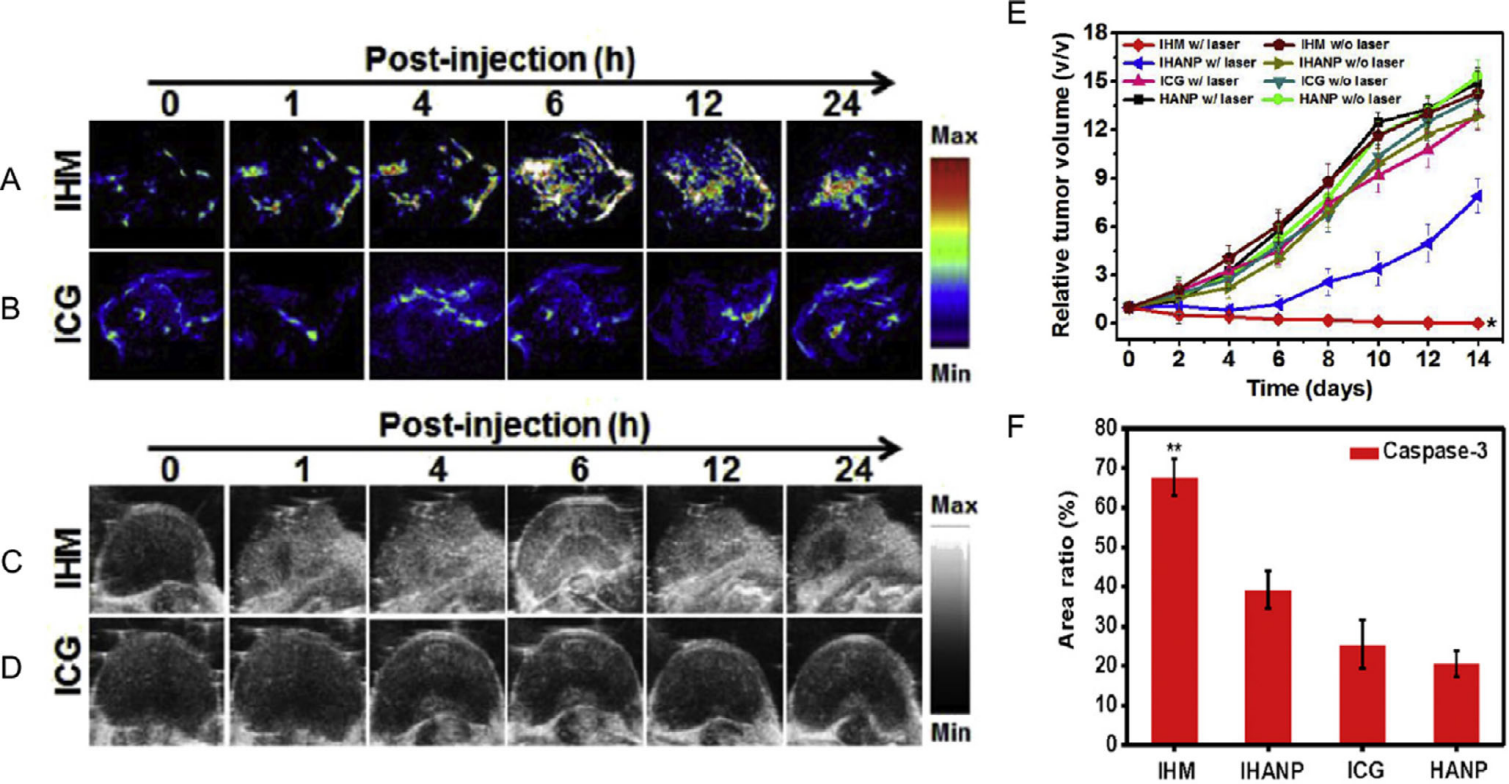
IHM‐mediated oxygen delivery for better PDT efficacy. (A and B) PA imaging of IHM and ICG densities in tumors. Maximal accumulation at the 6‐h mark. (C and D) Corresponding US image displayed on a gray colormap. Enhanced US signal was observed only in the IHM group and not in the ICG group. (E) Tumor volume reduction is the most effective in the IHM w/laser group when compared to other treatment groups, and (F) corresponding expression of caspase‐3, indicative of apoptosis‐mediated tumor death following PDT, calculated from histological analysis. Gao *et al*. reported IHM‐treated tumors were cured at 14‐days, with no recurrence by 30 days. Adapted with permission from 107.

Another development in the field of cancer therapeutics for enhancing PDT efficacy is the artificial presentation of exogenous oxygen to tumors. This may seem counterintuitive to the desired therapeutic endpoint of tumor destruction, as greater oxygen availability may lend itself to greater ROS generation by the tumor and thus an increase in cell division cycles and tumor progression. Conversely, shortage of oxygen may send the tumor down a path to hypoxia thus treatment resistance and enhanced metastases 97. The balance between these two avenues is indeed complex 106; however, many studies have shown that the availability of additional oxygen enhances PDT efficacy and thereby greater tumor destruction and better therapeutic endpoint. The PDT process consumes oxygen in a given tissue, the replenishment of which is dependent on local vascular perfusion. If vasculature is destroyed during PDT and the tumor is insufficiently affected, then both the availability of future molecular O_2_ for PDT‐mediated ROS generation and tumor hypoxia may result. By supplying additional oxygen to the area undergoing PDT, the conversion of molecular O_2_ to ROS may be enhanced, and the therapeutic breadth of PDT increased. Gao *et al*. reported a nanoparticle (IHM) containing indocyanine green and hyaluronic acid (HA) in its membrane, which conferred specific targeting to tumors that overexpress the HA receptor CD44 on their surface 107. Within the core of IHM is housed magnesium oxide, which generates O_2_ when it encounters H_2_O_2_ (hydrogen peroxide) (Fig. 10), the levels of which are aberrantly high in cancer cells 108. The resulting available O_2_ for conversion to ROS was increased two‐fold using Gao *et al*.’s nanoparticle, and ICG provided contrast for PA imaging. One shortfall to the IHM nanoparticle is that Gao *et al*. found it localizing to liver tissues, which was expected as it expressed a HA receptor, but is an important consideration for assessing the localization of PSs to nontarget tissues. Gao *et al*. proposed that this may be addressed by the addition of polyethylene glycol (PEG) to their nanoparticle to confer a more immunologically inert surface. Luo *et al*. developed a similar approach, but instead utilized hemoglobin‐ICG in a PLGA core with a lecithin/DSPE‐PEG outer membrane, dubbed I‐ARCs 109. In this modality, hemoglobin is converted to ferryl‐Hb after photodynamic illumination, providing a highly toxic and spatiotemporally specific agent for tumor destruction. Luo *et al*. reported complete remission of MCF‐7 tumors and no recurrence at 30 days post‐treatment**.** The balance of redox dynamics between normal and cancerous tissues is a complicated affair, and great care must be taken to understand the setting in which these treatments are applied in order to avoid fueling tumor hypoxia, growth or metastasis. Avoiding immunological degradation is a major hurdle for NP‐based therapies, and although PEGylation can make a nanoparticle more immunologically “stealthy,” cases of anti‐PEG immune responses have been reported in mammals 110. To avoid this, the exciting domain of red blood cell membrane‐derived nanoparticles (RBCM‐NPs) was developed. By creating these NP formulations with blood extracted from patients, several native cell surface receptors of RBCs are already present on these NPS and can aid in avoiding patient‐specific immunological responses. This corresponds to greatly improved circulation times, which can reach up to 120 days. Xia *et al*. reported on a number of developments in this arena where patient‐specific RBC character can be mapped onto the RBCM‐NP theranostic agents 111. Further preclinical testing of such novel theranostic nanoagents for US and PA image‐guided PDT is currently underway by several groups. Given the rate at which interdisciplinary collaborations between imaging and nanotechnology research groups is accelerating in both academia and industry, we anticipate the clinical translation of these nanotheranostic agents for PDT applications to come to fruition within the next few decades.

## Conclusions and Future Directions

PDT has tremendous potential for being at the forefront of the therapeutic battle against cancer. However, it is currently restricted to being an adjunct therapy or is employed for palliative care 5 due to hurdles such as the phototoxicities associated with the earlier generation of PSs and the unavailability of accurate dosimetry design strategies. With the advent of novel imaging technologies, nanotechnology‐enabled PS drug delivery, clearer understanding of the macroscopic and microscopic mechanisms involved in PDT, and the availability of light fiber delivery systems and dosimetry tools, PDT is well on its way to becoming a primary clinical treatment modality. In this review, we present the essential role that US and PA imaging could play in catapulting PDT to the forefront of cancer therapy. We discussed the utility of these imaging techniques in structurally and functionally characterizing tumors throughout the PDT pipeline, the design and deployment of optical fiber‐based modalities, the monitoring of tumor oxygenation status pre‐, during, and post‐PDT, and finally image‐guided enhanced PDT‐enabling nanotechnologies.

The low‐cost, mobile, high‐throughput, real‐time acquisition, noninvasive and nonionizing nature of US imaging has made it a key modality to monitor tumor volume. The availability of various clinically approved transducers such as the endoscopic, endocavity, endovaginal, pencil and transesophageal US probes enables the imaging of tumors in the internal structures or organs such as the pancreas, digestive tract and prostate. Only a handful of clinical studies exist that compared the performance of USI and MRI, the other nonionizing imaging modality also used to measure tumor volume and stage cancer. For example, Kim *et al*. showed that endoscopic US (EUS) imaging had a similar sensitivity, specificity and accuracy to MRI (statistically insignificant; *P*‐value > 0.05) in pancreatic cysts 112. The study by Fernández‐Esparrach *et al*. in 90 patients with rectal cancer concluded that there was no statistically significant difference in staging the disease between both MRI and EUS 113. Pinkavova *et al*. monitored the size of locally advanced cervical cancer following neoadjuvant chemotherapy and reported that the diagnostic accuracy for predicting tumor volume was similar to MRI; however, MRI showed higher sensitivity than US imaging in this study 114. In another study on 39 breast cancer patients by Lee *et al*., no significant difference in volume estimation by US imaging or MRI was observed; however, MRI had marginally higher (18.2%) predictive capability than US imaging in evaluating pathologic complete response in patients post‐treatment 115. Furthermore, aspects such as technical accuracy, clinician’s belief, and the necessity of pursuing surgical resection also factors into the wide adaptability of US imaging as discussed in a notable study conducted by Spolverato *et al*. 116. A major drawback of US imaging in accurately determining the size of a lesion is due to the hypothetical sound velocity used to calculate backscattered echo round trip time in image reconstruction. Since the velocities vary between various tissues (about 13%), an over‐ or underestimation of thickness as well as image aberrations could occur and several studies are currently being pursued to resolve this issue via correction of speed of sound values 117, 118. Another major drawback is identification of lesion boundaries leading to interoperator variability. Research on various machine learning, artificial intelligence and 3D reconstruction algorithms that can enhance the detection of tumor boundaries and reduce operator variability is currently being pursued which will further the utility of USI in PDT treatment monitoring 119.

While US imaging is ubiquitously available for patient care, PA imaging is also making strides in the clinical realm in characterizing prostate, breast and skin cancer lesions 120. As with US imaging, the frequency of the transducer is a major determining factor of resolution in PA imaging. Both in the preclinical and more recently in the clinical realm, PA imaging has gained tremendous popularity due to its capability to measure blood oxygen saturation in 3D at high penetration depth and resolution 69. Though the blood oxygen saturation and partial pressure of oxygen cannot be directly correlated and can generally be described via the Severinghaus equation 121, oxygen saturation maps have shown good correlation with cellular hypoxia markers in several preclinical and clinical studies 69, 122. For example, Gerling *et al*. showed good correlation between oxygen saturation and immunohistological hypoxia marker pimonidazole (*R* ^2^ = 0.887) 123, while a study by Tomaszewski *et al*. showed a moderate correlation (*R* ^2^ = 0.46) with cellular hypoxia marker carbonic anhydrase 124. In a recent clinical study on breast cancer patients, oxygen saturation levels were not statistically different between the carbonic anhydrase positive and negative cases; however, the total vascular perimeter showed a moderate correlation (*R* ^2^ = 0.43) with the oxygen saturation level only in the group that had received therapy previously 125. To obtain accurate oxygenation values, robust phantom and *ex vivo* and *in vivo* evaluations and comparisons between partial pressure of oxygen at the cellular level and blood oxygen saturation at the vascular level must be conducted. Moreover, strategies involving light delivery to deeper tissues, PA signal detection and signal processing and unmixing in the presence of additional NIR dyes such as PSs are being explored to improve accuracy of estimating blood oxygen saturation as recently reviewed by Li *et al*. 69 and Cao *et al*. 122. These research efforts and technological advances in the PA imaging realm are bound to make it a leading technique for designing and monitoring PDT.

Given the recent developments in US and PA imaging technology, including efforts to build low‐cost systems 126 and integrated theranostic setups, in the near future we can expect to see a combined US, PA and photodynamic probe that can image the pretreatment tumor condition, adjust PDT dose according to tumor vascular status, oxygenation status and PS concentration in the lesion, and monitor the therapy while PDT action is ongoing. With the advent of better light delivery system, faster data acquisition (by way of high repetition frequency lasers), better image processing algorithms, and transducers with better sensitivity, provides a strong foundation upon which it can be envisioned that the efforts on personalized US imaging and PA‐guided PDT treatment strategies will continue advancing the improvement of clinical outcomes in cancer.
